# Supplementary material for: Cytoophidium complexes resonate with cell fates
Source: Cell Mol Life Sci. 2025 Jan 21;82(1):54. doi: 10.1007/s00018-025-05578-z (PMC11751279; doi:10.1007/s00018-025-05578-z)
Supplement: Supplementary file 1 — Supplementary Material 1 [file 18_2025_5578_MOESM1_ESM.docx]

**Cytoophidium complexes resonate with cell fates**

**Yi-Lan Li^1^ and Ji-Long Liu^1,2,^***

**Supplementary info**

- **Supplementary Tables S1-S5**
- **Supplementary Figures S1-S6**
- **Supplementary Tables S1-S5**

**Supplementary Table S1. The strains used in this study.**

| Strain | Genotype | Description |
| --- | --- | --- |
| UGPA | BY4741 MATa his3Δ1 leu2Δ0 met15Δ0 ura3Δ0 URA7-mGFP:HIS3 GLT1-mCherry:LEU2 PRS5:mTagBFP:URA3 ASN1-miRFP670nano:KanMX | Control strain |
| GAUP | BY4741 MATa his3Δ1 leu2Δ0 met15Δ0 ura3Δ0 URA7- mTagBFP:URA3 GLT1- mGFP:HIS3 PRS5- miRFP670nano:KanMX ASN1- mCherry:LEU2 | Control strain backup |
| CTPS mutant | BY4741 MATa his3Δ1 leu2Δ0 met15Δ0 ura3Δ0 URA7(H360A, D370A, R391A, W392G)-mGFP:HIS3 URA8(H360A, D370A, K391A, W392G)-miRFP670nano:URA3 | Disruption the formation of cytoophidia for both CTPS, Ura7p and Ura8p. |
| CTPS mutant + Glt1 mutant | BY4741 MATa his3Δ1 leu2Δ0 met15Δ0 ura3Δ0 URA7(H360A, D370A, R391A, W392G)-mGFP:HIS3 URA8(H360A, D370A, K391A, W392G)-miRFP670nano:URA3 GLT1(H1537A, Y1538N, L1539S)-mCherry:LEU2 | Disruption the formation of cytoophidia for both CTPS and glutamate synthase Glt1p. |
| CTPS mutant with BNA5-mTB | BY4741 MATa his3Δ1 leu2Δ0 met15Δ0 ura3Δ0 URA7(H360A, D370A, R391A, W392G)-mGFP:HIS3 URA8(H360A, D370A, K391A, W392G)-miRFP670nano:URA3 BNA5-mTagBFP:KanMX | Visualizing BNA5 in the background of CTPS mutant. |
| CTPS mutant + Glt1 mutant with BNA5-mTB | BY4741 MATa his3Δ1 leu2Δ0 met15Δ0 ura3Δ0 URA7(H360A, D370A, R391A, W392G)-mGFP:HIS3 URA8(H360A, D370A, K391A, W392G)-miRFP670nano:URA3 GLT1(H1537A, Y1538N, L1539S)-mCherry:LEU2 BNA5- mTagBFP:KanMX | Visualizing BNA5 in the background of CTPS mutant + Glt1 mutant. |
| CTPS mutant OE | BY4741 MATa his3Δ1 leu2Δ0 met15Δ0 ura3Δ0 URA7(H360A, D370A, R391A, W392G)-mGFP:HIS3 URA8(H360A, D370A, K391A, W392G)-miRFP670nano:URA3 pL60K-CEN/ARS-pURA7>URA7(H360A, D370A, R391A, W392G)-mGFP:KanMX | Overexpression of URA7 mutant in the background of CTPS mutant. |
| CTPS mutant + SIR2 OE | BY4741 MATa his3Δ1 leu2Δ0 met15Δ0 ura3Δ0 URA7(H360A, D370A, R391A, W392G)-mGFP:HIS3 URA8(H360A, D370A, K391A, W392G)-miRFP670nano:URA3 pL70K-CEN/ARS-pSIR2>SIR2:KanMX | Overexpression of SIR2 in the background of CTPS mutant. |
| BNA5 KO | BY4741 MATa his3Δ1 leu2Δ0 met15Δ0 ura3Δ0 URA7-mGFP:HIS3 GLT1-mCherry:LEU2 bna5Δ::URA3 | BNA5 knockout strain with URA7 and GLT1 tagged by fluorescent proteins. |

All strains were initially constructed in this study.

**Supplementary Table S2. The primers used in this study.**

| Primer | Sequence 5’🡪3’ | Description | Template |
| --- | --- | --- | --- |
| Pu7-rc-F | TAGTTCCTGTCATCCACGGTG | URA7-mGFP:HIS3 knock-in | L02+ |
| Pu7g-rc-R | TTGTTAATGCAGTAGACTTTTAATTCTAAAATTTTGATCAGTATAGCGACCAGCATTCAC |  |  |
| Glt1-mc-F59 | ACGTGATTACAAACTATTGAAAGAATTAGCTAGTCAAGTCGGAGGTAGCGGTAGCAAGGGCGAGGAGGATA | GLT1-mCherry:LEU2 knock-in | L04 |
| Leu2-GLT1-R59 | AAATAATATACGATCATAAAATAAATAATAACTCAAGCTTTTGTGGTGCCCTCCTCCTTG |  |  |
| Asn1-L13-F58 | TGCTGAAGATCCTTCTGGTAGATATGCCCAAATTCATGAAAAACATATCGAAggtgGTAGCAGCGGAT | ASN1-miRFP670nano:KanMX knock-in | L13 |
| Asn1-L13-R58 | AAATATCTATAAGATTAATCCATAATTCTTTTTCTATTTTTTAATGTTATGCAGTATAGCGACCAGCATTC |  |  |
| Prs5-mtBFP-F60 | TAACGGTGAATCCATTTCTATGTTGTTCGAGCATGGATGGATTGGTAGTGGGAGCAACGG | PRS5-mTagBFP:URA3 knock-in | L08 |
| Ura3-prs5-R58 | CCCTATTTTTATCAATAAAAAAATGAACACATCAATGCCATTAGTTTTGCTGGCCGCATCT |  |  |
| Pu7MUT-D-F | TCCATGAAGTGTCGTCGTAAG | URA7-mutant:mGFP knock-in | L37.5 |
| Pu8MUT-F60b | ACGTACCACGTTCCCTTATTGCTG | URA8-mutant-miRFP670nano:URA3 knock-in | L51 |
| URA8-URA3-R59 | ATAATATACTTGAATAAAAACCGTATGTAAGTAAGTAGTAAGCGCATCAATGCGGTATTTCACACCGCAG |  |  |
| GLT1-1292-F58 | CCACACCCGTCTAGATAATAAGTTAATCG | GLT1-mutant-mCherry:LEU2 knock-in | L57 |
| BNA5-mTB-F59 | AGATGTATACATTGCGGTGAATGCACTAAATGAGGCGATGGATAAGTTGggcattggtagtgggagca | BNA5-mTagBFP:KanMX knock-in | L61 |
| BNA5-miR670n-R59 | TCATATATAATATCCAAAAGAAGATGAAGGCGATGCGGTCACTCTAGGCAGTATAGCGACCAGCATTCAC |  |  |
| BNA5-URA3-F59 | GTCATATATAATATCCAAAAGAAGATGAAGGCGATGCGGTCACTCTAGGAATTGTACTGAGAGTGCACCACG | bna5Δ::URA3 knock-out | L08 |

**Supplementary Table S3. The plasmids used in this study.**

| Plasmids | Genotype | Description |
| --- | --- | --- |
| L02+ | pL02-URA7-mGFP(S65T, A206K)-ADH1ter-pTEF>HIS3-TEFter-AmpR | Used for mGFP tagging in genome. |
| L04 | pL04-CEN/ARS-mCherry-ADH1ter pLEU2>LEU2-AmpR | Used for mCherry tagging in genome. |
| L08 | pL08-CEN/ARS-mTagBFP-ADH1ter pURA3>URA3-AmpR | Used for mTagBFP tagging in genome. Used for gene replacement by URA3 as well. |
| L13 | pL13-CEN/ARS-pGAP>LifeAct-miRFP670nano-ADH1ter-KanMX-AmpR | Used for miRFP670nano tagging in genome. |
| L37.5 | pL37.5-URA7(H360A, D370A, R391A, W392G)-mGFP-ADH1ter-pHIS3>HIS3-AmpR | Used for URA7 endogenous gene mutation. |
| L51 | pL51-URA8(H360A, D370A, k391A, W392G)-miRFP670nano-ADH1ter-pURA3>URA3-AmpR | Used for URA8 endogenous gene mutation. |
| L57 | pL57-CEN/ARS-GLT1(H1537A, Y1538N, L1539S)-mCherry:LEU2-AmpR | Used for Glt1 endogenous gene mutation. |
| L60K | pL60K-CEN/ARS-pURA7>URA7(H360A, D370A, R391A, W392G)-mGFP:KanMX-AmpR | Used for URA7 mutant overexpression |
| L61 | pL61-CEN/ARS-mTagBFP-ADH1ter KanMX-AmpR | Used for mTagBFP tagging in genome, but with a KanMX selection |
| L70K | pL70K-CEN/ARS-pSIR2>SIR2:KanMX-AmpR | Used for SIR2 overexpression |

All plasmids were initially synthesized in this study.

**Supplementary Table S4. Reagents.**

| Reagents | Brand | Cat Number | Description |
| --- | --- | --- | --- |
| Phanta Max Super-Fidelity DNA Polymerase | Vazyme | P505 | PCR |
| 2X MultiF Seamless Assembly Mix | ABclonal Technology | RK21020 | Gibson assembly |
| Lithium acetate dihydrate | Macklin | L812345 | Yeast transformation |
| PEG4000 | Meilunbio | MB2587 | Yeast transformation |
| TE (Tris/EDTA buffer) | Beyotime Biotechnology | ST725 | Yeast transformation |
| ssDNA | Sigma-Aldrich | D9156 | Yeast transformation |
| DpnI | ABclonal Technology | RK21109 | Template digestion |
| PCR cleanup kit | UElandy | UE-PCR-250 | PCR cleanup |
| Nicotinic acid | Accela ChemBio | SY011111 | Chemical addition |
| Nicotinamide | Accela ChemBio | SY024804 | Chemical addition |
| Cytosine | Accela ChemBio | SY001643 | Chemical addition |
| Yeast Extract | Oxoid | LP0021B | Yeast culture |
| Peptone | Sigma-Aldrich | 82962 | Yeast culture |
| Glucose | Solarbio | G8150 | Yeast culture |
| YNB w/o AA or (NH_4_)_2_SO_4_ | BBI | A600505 | Yeast culture |
| Succinic acid | Collins | P2819863 | Yeast culture |
| Ammonium sulfate | Sinopharm Chemical Reagent | 10002918 | Yeast culture |
| Tyrosine | Sigma-Aldrich | T8566 | Yeast culture |
| Methionine | Sigma-Aldrich | M5308 | Yeast culture |
| Arginine | Sigma-Aldrich | A5006 | Yeast culture |
| Alanine | Sigma-Aldrich | A7469 | Yeast culture |
| Serine | Sigma-Aldrich | S4311 | Yeast culture |
| Valine | Sigma-Aldrich | V0513 | Yeast culture |
| Threonine | Sigma-Aldrich | T8841 | Yeast culture |
| Isoleucine | Sigma-Aldrich | I7403 | Yeast culture |
| Glycine | Sigma-Aldrich | G8790 | Yeast culture |
| Phenylalanine | Sigma-Aldrich | P5482 | Yeast culture |
| Cysteine | Sigma-Aldrich | C7352 | Yeast culture |
| Aspartic acid | Sigma-Aldrich | 17219 | Yeast culture |
| Glutamic acid | Sigma-Aldrich | G1626 | Yeast culture |
| Proline | Sigma-Aldrich | P5609 | Yeast culture |
| Tryptophane | Sigma-Aldrich | T8941 | Yeast culture |
| Leucine | Sigma-Aldrich | L8912 | Yeast culture |
| Histidine | Sigma-Aldrich | H5659 | Yeast culture |
| Uracil | Sigma-Aldrich | U1128 | Yeast culture |
| Lysine | Sigma-Aldrich | L8662 | Yeast culture |
| G418 | GPC | AK108 | Yeast culture |
| Sodium hydroxide | Sinopharm Chemical Reagent | 10019718 | Yeast culture |
| Agar | Abcone | A42307 | Yeast culture |
| Fluorescent Brightener 28 disodium salt solution (CFW) | Sigma-Aldrich | 910090 | Imaging |
| Low gelling point agarose | Sigma-Aldrich | A4018 | Imaging |
| Formalin | Sigma-Aldrich | F8775 | Imaging |
| Dihydroethidium | SparkJade | SJ-MD0019 | Imaging |
| Percoll | Yeasen | 40501ES60 | Cell separation |
| Sodium chloride | Sinopharm Chemical Reagent | 10019318 | Cell separation |
| 20x PBS buffer | Sangon Biotech | B548117 | Buffer |
| TransZol Up | Transgen | ET111-01 | RNA extraction |

**Supplementary Table S5. Materials.**

| Materials | Brand | Cat Number | Description |
| --- | --- | --- | --- |
| 9cm petri dish | PULLEN | PX02003 | Yeast culture |
| 35mm Glass Bottom Dish | MatTek | P35G-1.5-10-C | Imaging |
| Cover slip | Brand | 470050 | Imaging |
| Adhesion Slide | Titan | SWBP-Z0003 | Imaging |
| Glass beads | Sigma-Aldrich | G8772 | RNA extraction |
| Syringe Filter, 0.22 µm | Millipore | SLGPR33RB | Sterilization |
| 13.2mL Open-Top Ultra-Clear Tube | Beckman Coulter | 344059 | Ultracentrifugation |

- **Supplementary Figures S1-S6 and figure legends**

**
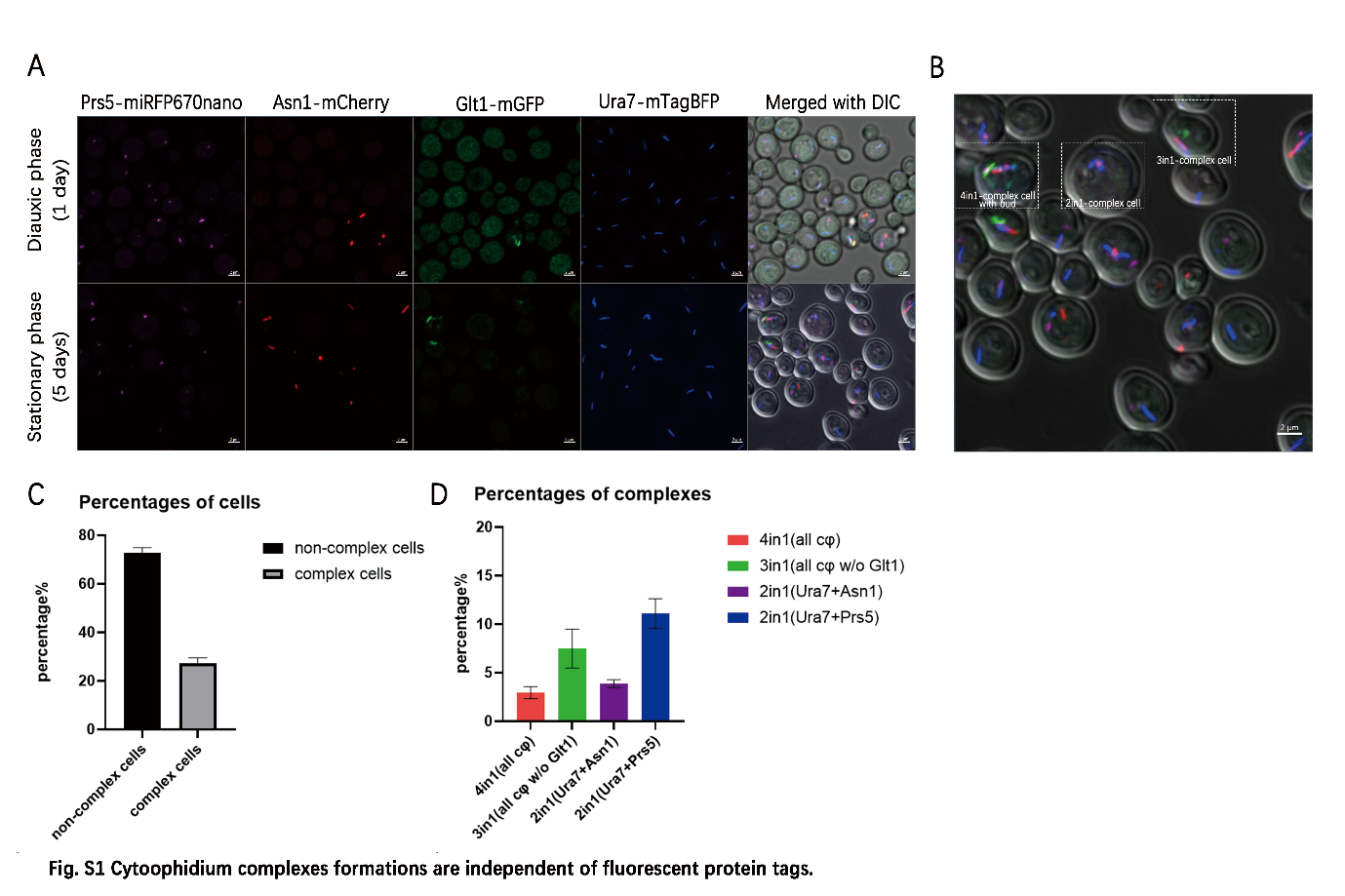
**

**Figure S1. The formation of cytoophidium complex is independent of fluorescent protein tags.**

**A.** The formation of cytoophidia and complexes during different growth stages. Purple represents Prs5-miRFP670nano, red represents Asn1-mCherry, green represents Glt1-mGFP, blue represents Ura7-mTagBFP. Scale bar = 2 μm. **B.** Zoom-in of stationary phase cells in A, and cells with different complexes are labelled by squares. Scale bar = 2 μm. **C-D.** Quantification of the percentage of cells containing or not containing cytoophidium complexes (C) and the percentage of complexes of different compositions (D).


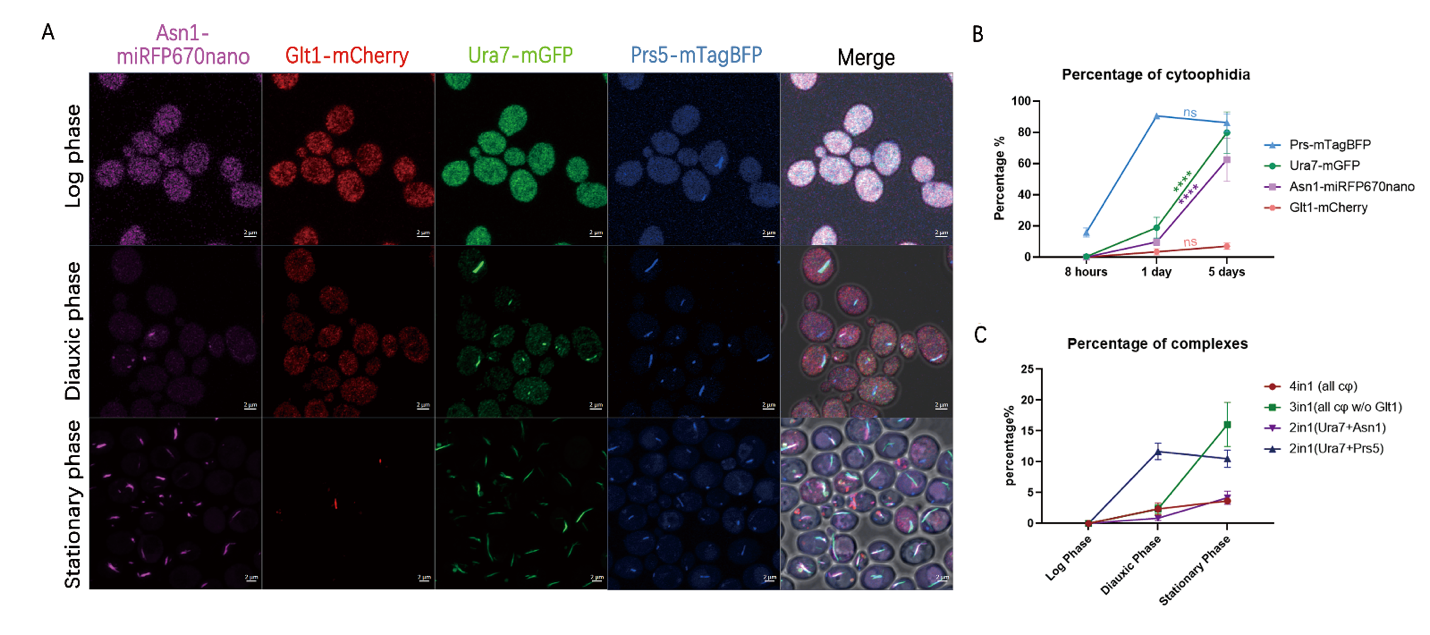


**Figure S2. Cytoophidium abundance and complex abundance during cultivation.**

**A.** The formation of cytoophidia and complexes during different growth stages. Purple represents Asn1-miRFP670nano, red represents Glt1-mCherry, green represents Ura7-mGFP, blue represents Prs5-mTagBFP. Scale bar = 2 μm. **B.** Quantification of the abundance of different cytoophidia during different growth stages. **C.** Quantification of the abundance of cytoophidium complexes with different compositions.


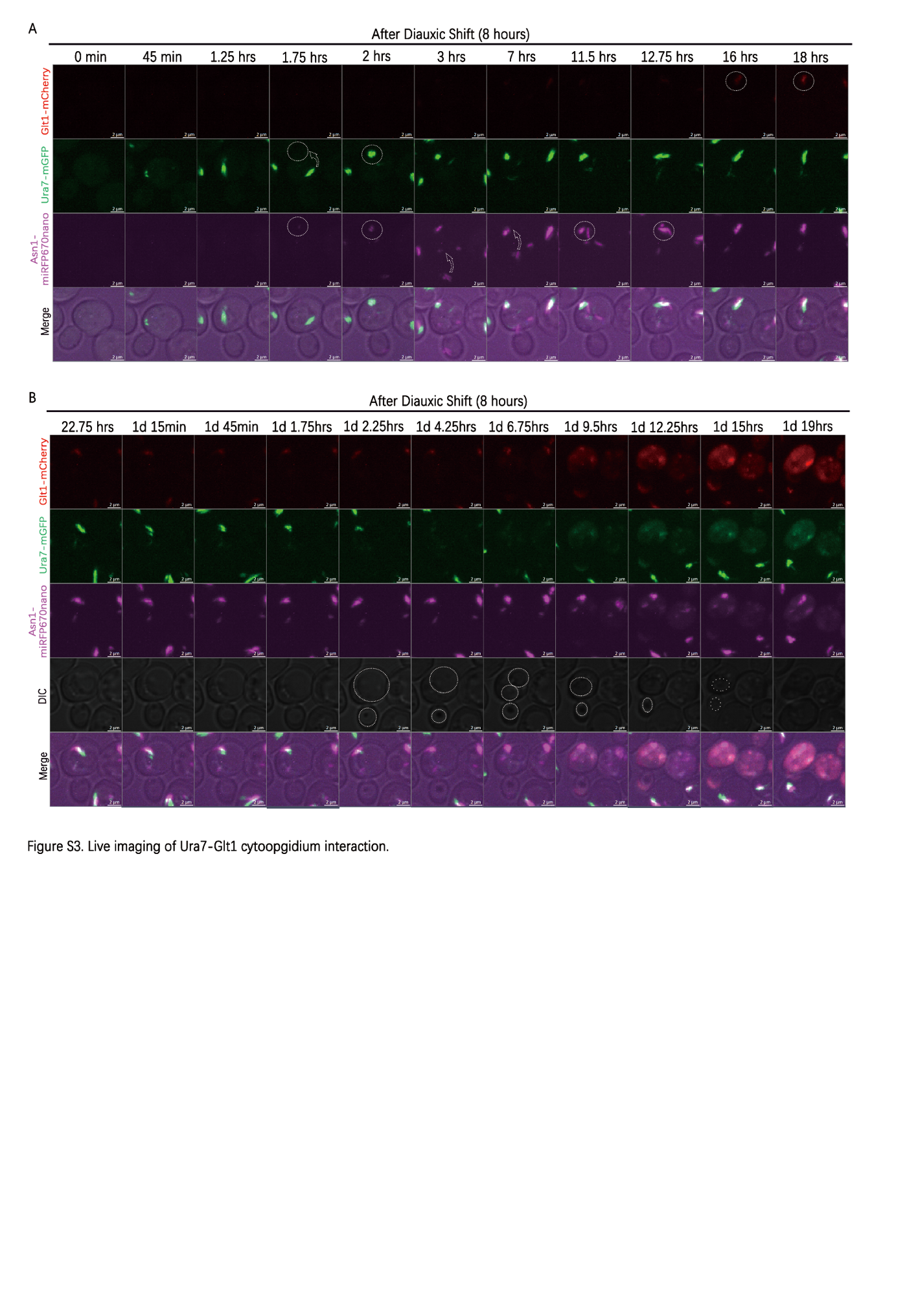


**Figure S3**. **Live imaging of Ura7-Glt1 cytoophidium interactions.**

**A.** The sequence of cytoophidium formation during normal culturing includes Ura7, Asn1, and Glt1 cytoophidium. Circles indicate areas of cytoophidium occurrence. Arrows indicate the movement of cytoophidia. **B.** Sequence of cytoophidium remodeling and cell shrinkage. Circles indicate the size of vacuoles in both mother and daughter cells. Scale bar = 2 μm.


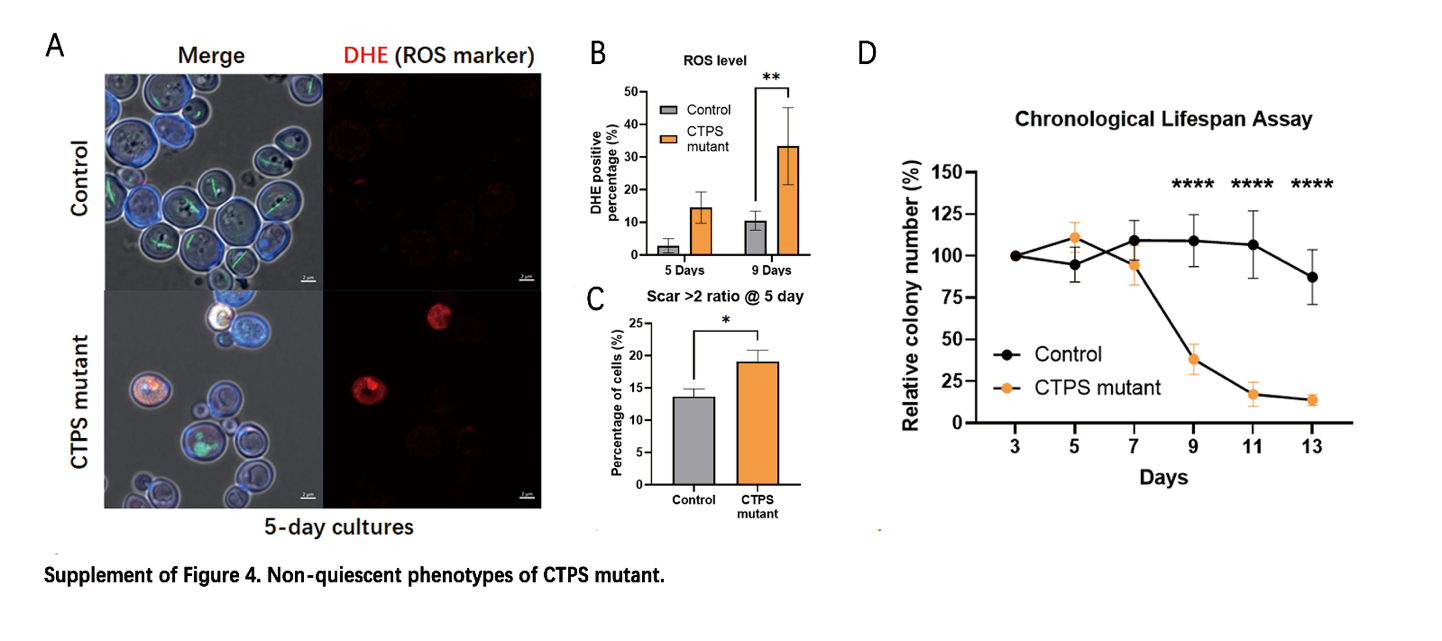


**Figure S4.** **The non-quiescent phenotype of CTPS mutant.**

**A.** Confocal images of ROS marker DHE (red) in Control strain and CTPS mutant at stationary phase (5 days). Ura7-mGFP (green), CFW (cell wall, blue), DIC (white). **B.** ROS level plot of strains in A at stationary phase (5 days and 9 days). **C.** The percentage of cells with 2 or more scars in the control strain and CTPS mutant at stationary phase (5 days). D. The chronological survival rate of the control strain and CTPS mutant from 3 days to 13 days. * means p-value < 0.05; **, p-value < 0.01; *** p-value < 0.001; ****, p-value < 0.0001; and ns or not labelled mean no significance in the statistical chart.


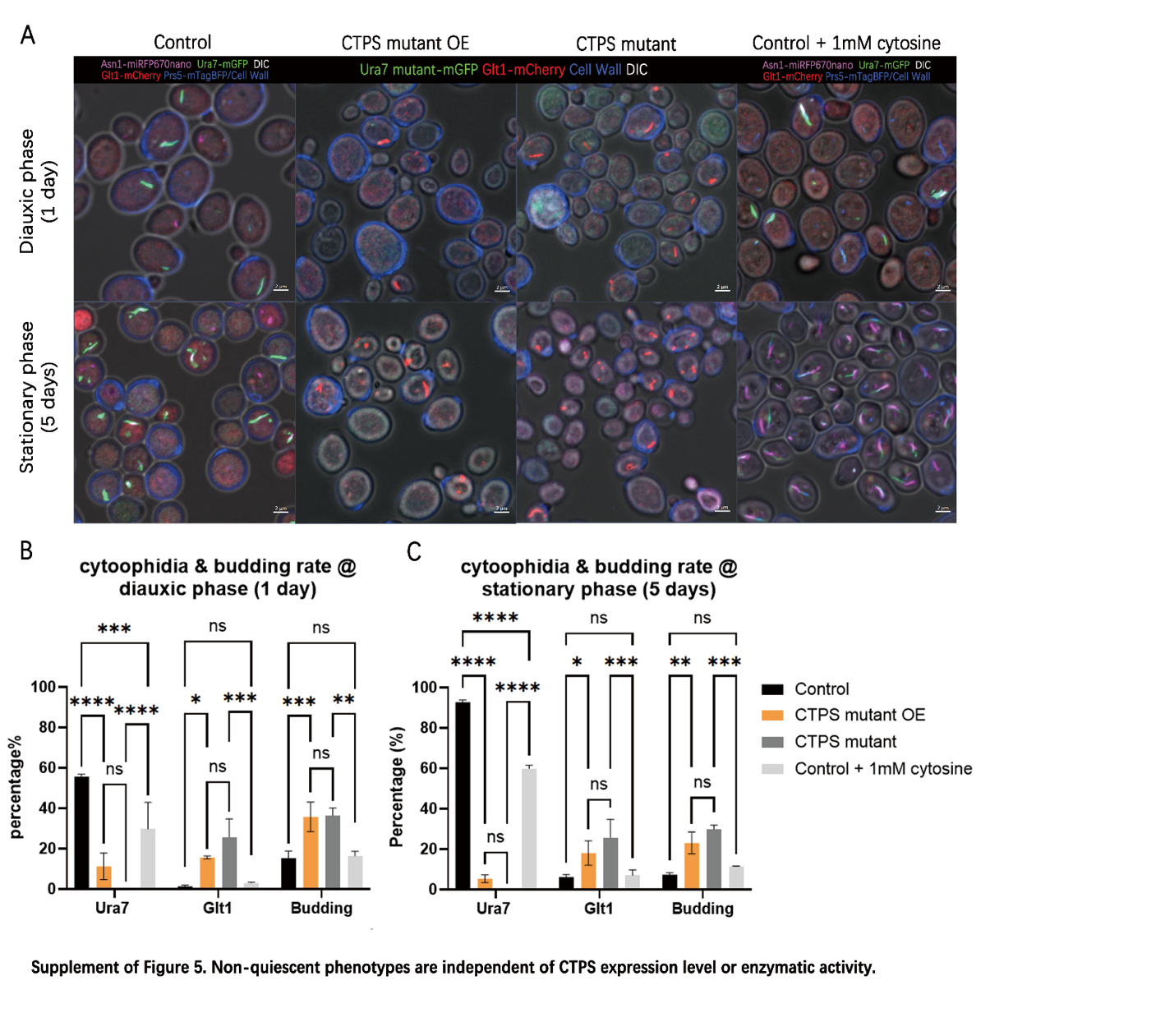


**Figure S5. The non-quiescent phenotype is independent of the expression level or enzymatic activity of CTPS.**

**A.** Confocal images of control strain (UGPA), CTPS mutant (Ura7^mutant^-mGFP, Ura8^mutant^-miRFP670nano, Glt1-mCherry), CTPS mutant OE (a URA7^mutant^-mGFP overexpression plasmid within CTPS mutant strain) and Control treated with 1 mM cytosine at diauxic phase (1 day) and stationary phase (5 days). For the control strain with complex, Ura7 signal (green), Glt1 signal (red), Prs5-mTagBFP /CFW (blue), Asn1-miRFP670nano and DIC (white) are shown. For the mutants, only Ura7 signal (green), Glt1 signal (red), CFW (cell wall, blue) and DIC (white) are shown. Scale bar, 2μm. **B,C.** Plots showing the percentages of Ura7 cytoophidium, Glt1 cytoophidium and budding rate in strains mentioned in A at diauxic phase (1 day, B) and stationary phase (5 days, C) . * means p-value < 0.05; **, p-value < 0.01; ***, p-value < 0.001; ****, p-value < 0.0001; and ns or not labelled mean no significance in the statistical chart.


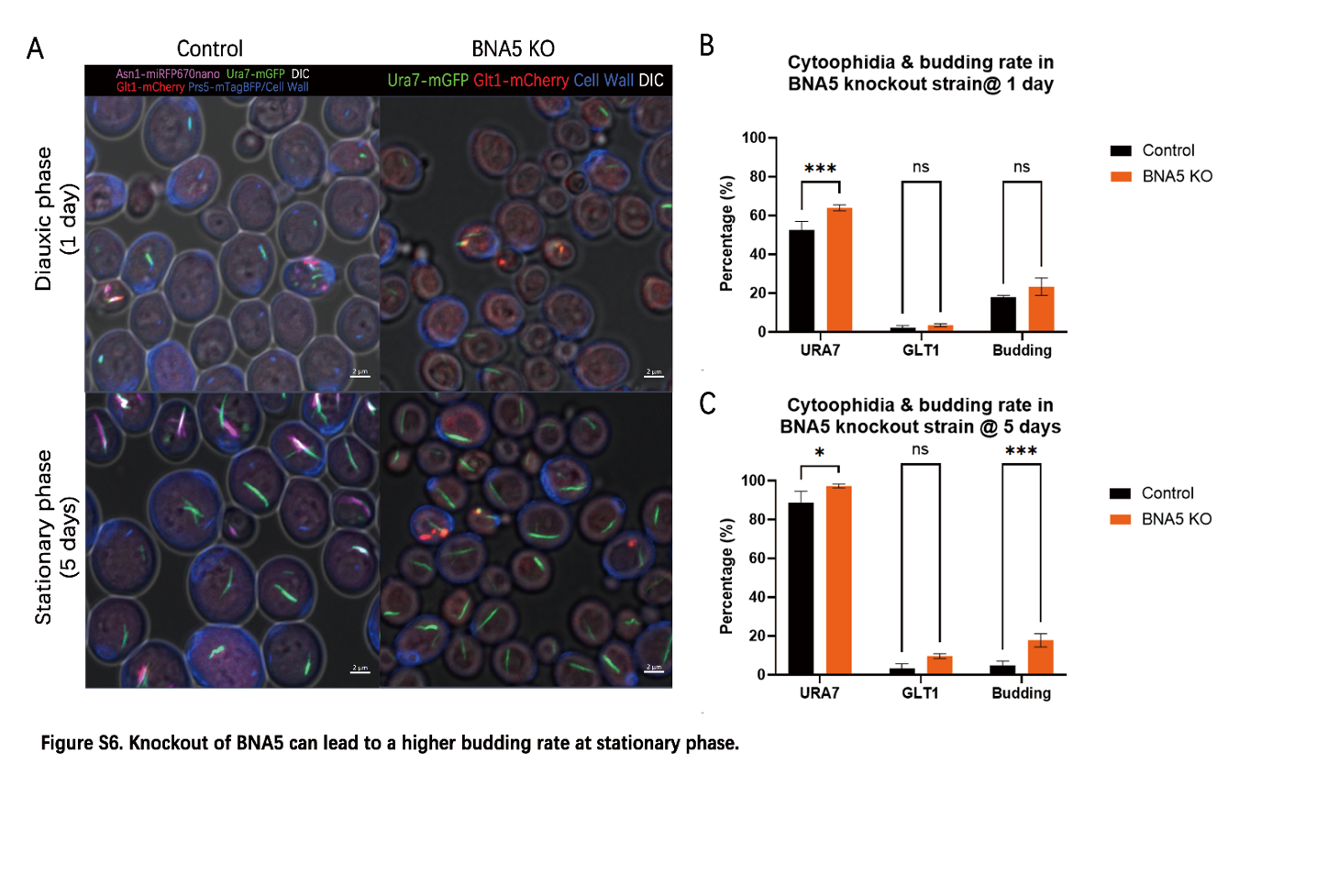


**Figure S6. Knockout of BNA5 can lead to a higher budding rate at stationary phase.**

**A.** Confocal images of control strain (UGPA) and BNA5 KO (with URA7-mGFP & Glt1-mCherry) at diauxic phase (1 day) and stationary phase (5 days). **B,C.** Quantification of URA7, GLT1 cytoophidia percentages among cells and the budding rate of the strains mentioned in A at 1 day (B) and 5 days (C). Scale bar = 2 μm. * means p-value < 0.05; **, p-value < 0.01; ***, p-value < 0.001; ****, p-value < 0.0001; and ns or not labelled mean no significance in the statistical chart.
